# Effects of Processing Conditions on Texture and Microstructure Evolution in Extra-Low Carbon Steel during Multi-Pass Asymmetric Rolling

**DOI:** 10.3390/ma11081327

**Published:** 2018-07-31

**Authors:** Satyaveer Singh Dhinwal, Laszlo S. Toth, Peter Damian Hodgson, Arunansu Haldar

**Affiliations:** 1 Laboratory of Excellence on Design of Alloy Metals for Low-Mass Structure (Labex-DAMAS), Université de Lorraine, 57070 Metz, France; 2 Université de Lorraine, CNRS, Arts et Métiers ParisTech, LEM3, F-57000 Metz, France; 3 Institute for Frontier Materials (IFM), Deakin University, Geelong 3216, Australia; peter.hodgson@deakin.edu.au; 4 Department of Metallurgy & Materials Engineering, Indian Institute of Engineering Science and Technology, Shibpur-711103, India; arunansuhaldar@gmail.com; 5 Tata Steel Ltd., Jamshedpur-831001, India

**Keywords:** rolling, asymmetric ratio, texture, thickness reduction per pass, grain refinement, steel

## Abstract

Multi-pass rolling was carried out on extra-low carbon steel at room temperature by imposing different ratios of asymmetry in the roll-diameters as well as by conventional mode. The aim of this study is to understand the effect of shear deformation due to the asymmetric conditions on the development of the rolling texture and the possibilities of propagating the shear deformation into the mid-thickness area of the sheet. The trends of the measured texture developments in both symmetric and asymmetric rolling indicate their dependence primarily on the stability and fraction of the Goss {110}<001> and the rotated cube {001}<111> orientations. The effects of asymmetry conditions were further examined on the microstructure evolution and were correlated to the increased orientation inhomogeneity and grain fragmentation. Both texture and microstructure development showed their dependence on the applied thickness reduction per pass, on the total thickness reduction of the sheet as well as on the degree of the imposed asymmetry. It was found that shear textures can be obtained by asymmetric rolling at conditions where all three parameters—asymmetry ratio, strain in one pass, and the total accumulated strain—are as large as possible.

## 1. Introduction

Asymmetric rolling is one of the most convincing alternatives for producing continuous sheets with through thickness shearing as compared to other existing shearing-based processes in sheet manufacturing [1,2,3,4,5,6,7,8,9,10]. The growing interest in this kind of shear based processing is due to the relatively simple modification that existing conventional (symmetric) rolling requires, just by varying a few rolling parameters while keeping the rest of the industrial sheet processing infrastructure almost unchanged. Various studies report that when an asymmetry is introduced in the roll-diameters of the rolling process leads not only to an increase in the productivity of sheet manufacturing but also enhances the formability of the product [11,12,13,14]. Such an increase in formability was related to the tilting/rotation of the conventional rolling texture by the imposed shearing, resulting from the asymmetric conditions. Investigations about the rotation of conventional rolling texture in asymmetric rolling further show its dependence on factors such as the thickness reduction per pass (TRPP), the type of applied asymmetry (kinematical, mechanical, or tribological), and the ratio of these imposed asymmetries applied to the two rolls [8,9,10,12,15,16,17,18,19,20,21,22].

Due to the various parameters that can be utilized for imposing asymmetry in rolling, different investigators applied different sets of rolling schedules. Based on the applied amount of thickness reduction per pass (TRPP), they can be classified into two categories. The small to medium type TRPP in the range of 5–50% are applied in multi-pass rolling while, on other hand, TRPP in the range of 60–75% are examined in a single pass. Results from these investigations provide varying ranges of tilting/rotation of the rolling texture and of its heterogeneity through the thickness. Some simulation-based and experimental studies suggest that the rotation of conventional rolling texture can reach close to the ideal shear texture with small TRPP of 5 to 15% [16,22]. Meanwhile, single pass rolling studies with a high TRPP of 60–75% were more concerned with grain refinement and mechanical properties than the possibilities of rolling texture rotation towards the ideal shear texture [13,18,23,24,25].

The current situation regarding asymmetric rolling schedules does not provide a clear understanding about the connection between TRPP, imposed asymmetry and the rotation of rolling texture or its trends through the thickness of the sheet. In addition, it can be noted from the literature on asymmetric rolling that there is a lack of experimental evidence about the behavior of prominent texture components of the rolling texture regarding their tilting/rotation imposed by the asymmetric conditions. Nevertheless, some simulation based studies predicted the overall nature of rolling textures with assumed shear coefficient values (the ratio of shear strain/rolling strain) [10,22,26,27].

It should also be noted that, apart from a very few studies on BCC materials, most experimental and simulation based studies were carried out on the HCP and FCC materials [16,21,22,28,29]. However, BCC metals form a very important class of structural materials and have a strong tendency to develop favorable textures, well suited for secondary processing when they are produced by conventional rolling. The potential of asymmetric rolling and its effect on this class of materials has not yet been fully addressed. An alteration in conventional texture by asymmetric rolling may lead to the future implications not only for structural applications but also for others. For example, asymmetric rolling can influence the magnetization properties of silicon steels. 

Existing knowledge about asymmetric rolling also does not clarify the respective influence of the shearing effect together with the number of passes and the total final thickness reduction of a sheet. A systematic study of multi-pass asymmetric rolling in comparison with symmetric rolling is indeed a requirement for understanding the characteristic texture and microstructural developments which also results in modification of the mechanical properties of the sheet. In the present study, an extra-low carbon steel was selected for the experiments. The following experimental parameters were varied: thickness reduction per pass (TRPP), total reduction, and the degree of applied roll diameter asymmetry. The employed total thickness reductions went up to very high values: to 89%. The resultant texture rotations and microstructural development were examined and related to the degree of shearing through the sheet. Special emphasis was given to the mid-thickness region since it is known that in the sub-surfaces of a sheet, ideal shear texture can readily form even under symmetric rolling conditions [30,31,32,33].

## 2. Materials and Methods 

**Figure 1:**
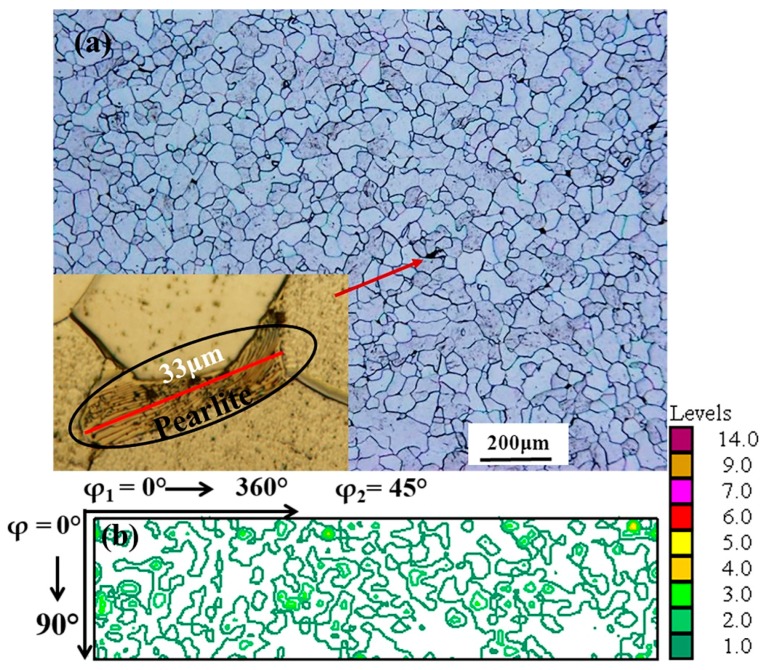
(**a**) Microstructure and (**b**) texture of extra-low carbon steel sheet after heat treatment, before rolling.

The nominal composition of the as-received extra-low carbon steel can be seen in Table 1. The plates were hot rolled at 1000 °C during which their thickness was reduced from 27 mm to 10 mm. After hot rolling, the sheets were heat treated for one hour also at 1000 °C and subsequently air cooled. This heat treatment was given to facilitate homogenization of the microstructure and to provide a weak initial texture (Figure 1). Apart from the ferrite phase, the co-existence of scattered tiny pearlite colonies can also be seen in Figure 1a. The average ferrite grain size and surface area fraction of pearlite colonies after the heat treatment were about 36 µm and less than 0.1%, respectively, measured by the open source ImageJ™ software on several optical micrographs. Samples with dimensions of 40 mm width, 70 mm length, and 8 mm in thickness were machined from the heat-treated sheets for the rolling schedules. The front ends of these samples were wedged for easier entry into the roll bite.

Rolling was carried out at room temperature with no lubrication in order to keep similar conditions as it had been in all previous reports on asymmetric rolling. However, it was important that the sheet material should not stick to the rolls. For this purpose, the rolls were lubricated after each pass and later cleaned with oil absorbing paper, so that the maximum amount of lubricant could be removed while retaining the smoothness of the rolls at constant conditions. Three roll diameter ratios 1:1.3, 1:1.6, and 1:2 were used to impose different degrees of asymmetric rolling, as compared with symmetric rolling (1:1). A special roll-set was designed for the experiments in order to facilitate all the ratios adjacent to each other on two shafts, both rotated at 32 revolutions per minute, driven by a single motor. The small diameter roll set was positioned and connected to the upper shaft of the mill while the counterpart large diameter roll set fitted to the lower shaft. TRPP values of 10% were used only for the ratios 1:1 and 1:2 while the 30% and 50% TRPP levels were applied for all roll diameter ratios. Rolling was unidirectional in all cases.

For texture measurements, X-ray diffraction from Panalytical X’pert Pro Instrument (PHILIPS, The Netherlands) was employed using a parallel beam point-focused on the specimen with its surface parallel to the rolling plane at the required thickness depth. The measured bulk texture results were analyzed using the commercially available Labosoft™ 3.0 software. The texture results are presented here in the $\varphi_{2}$ = 45° sections of the orientation space where almost all prominent rolling and shear texture components can be seen. Note that positions of shear texture components in the $\varphi_{2}$ = 45° section correspond to simple shear with shear plane in the plane of the sheet and with shear direction parallel to the rolling direction. The identifications of the ideal orientations are given with their Miller indices for rolling in Table 2 and for shear in Table 3. Because of the asymmetric deformation conditions, the texture analysis was carried out without imposing any kind of sample symmetry. This is why the equivalent orientations for symmetric rolling (f1–f2, e1–e2) are specified separately in Table 2 (they are not equivalent when shear is present, due to the loss of orthorhombic symmetry). To quantify the volume fraction of the main texture components, the orientation density function (ODF) was integrated within 10° orientation distance from the ideal position.

The microstructures were examined by the electron back scattered diffraction (EBSD) technique using a Zeiss LEO 1530 field emission gun scanning electron microscope (FEG-SEM). All samples were scanned in the mid-thickness area of the TD plane. The EBSD data acquisition and post-processing were performed by the AZTEC and HKL channel 5 software provided by Oxford Instruments. For the representation of the microstructures, inverse pole figure (IPF) maps were used where the color key code corresponds to the orientation of the RD axis with respect to the crystal coordinate system. Grain boundaries were identified on the basis of a minimum misorientation angle of 5°. Nevertheless, for a clear visualization, only grain boundaries with ≥15° misorientations were traced on the IPF maps. The misorientations, grain boundaries, and grain size distributions of the EBSD data were analyzed by the ATEX software [34].

**Figure 2:**
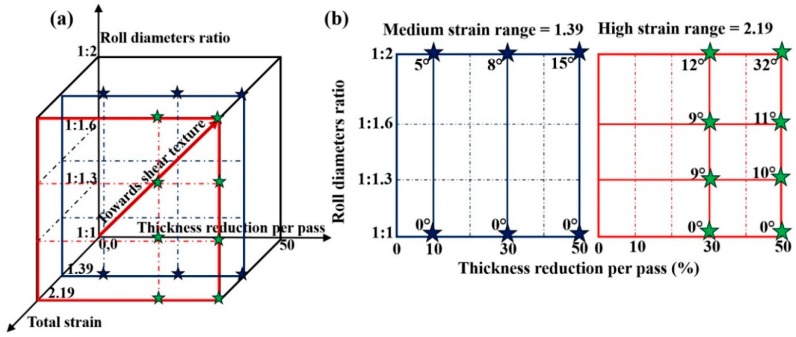
Schematic of the parameter space (**a**) and two sections (**b**) showing the parameter values employed in the present study. The numbers in (**b**) indicate the rotation angle of the rolling texture towards a shear texture, which is identified by the rotation of the f2 rolling component.

There are several parameters that can be varied for asymmetric rolling. As mentioned in the Introduction, three of them were selected for the present experiments: TRPP, total reduction and the degree of applied asymmetry. Figure 2 displays the parameter space in a 3D representation and also in two sections; at constant rolling strains of 1.39 and 2.19. The stars indicate the different combinations of the selected parameters and corresponding rotation of texture achieved by these combinations of parameters. There were 14 cases studied in total, most of them for large parameter values.

## 3. Results

### 3.1. Texture

#### 3.1.1. Medium Strain Range (75%)

**Figure 3:**
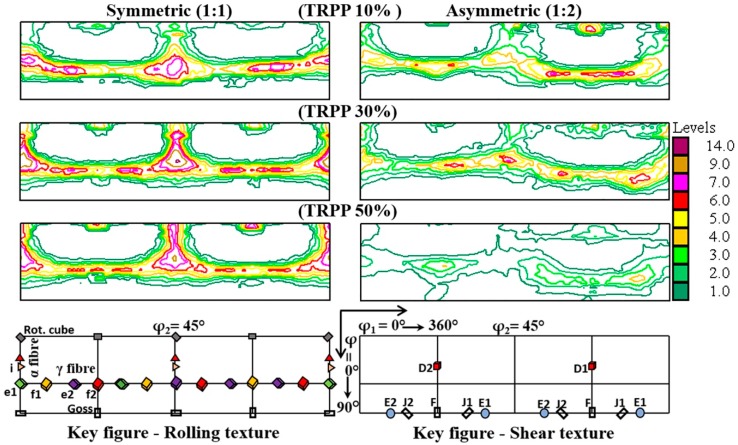
φ_2_ = 45° ODF sections at mid-thickness for symmetric (1:1) and asymmetric (1:2) rolling in medium strain range (75%) with TRPP of 10, 30, and 50%.

Figure 3 represents the φ_2_ = 45° sections of the ODF for the multi-pass rolling schedules for symmetric (1:1) and asymmetric (1:2) modes with TRPP varying between 10% and 50%. It can be seen in Figure 3 that the resultant texture remains almost identical to the typical symmetric rolling texture after asymmetric rolling with roll diameter ratio of 1:2 and 10% TRPP. Figure 3 also shows that while keeping the same asymmetric rolling ratio, both the intensity of the texture and its fiber nature decreases as the TRPP increases. 

**Figure 4:**
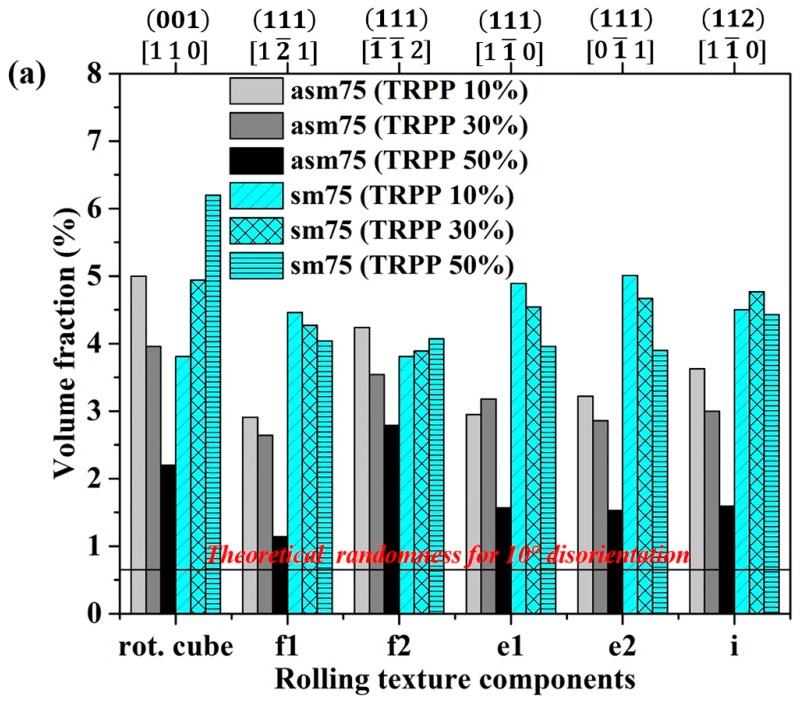

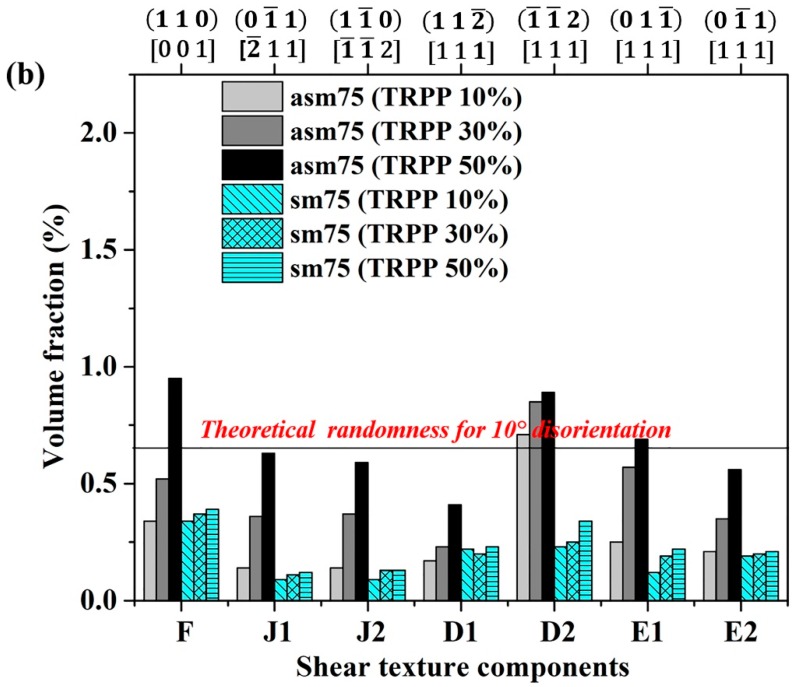
Volume fractions of the prominent (**a**) rolling and (**b**) shear texture components at mid-thickness in multi-pass symmetric and asymmetric (1:2) rolling in medium strain range (75%).

The volume fractions of the prominent rolling and shear texture components are presented in Figure 4 after 75% total thickness reduction as a function of TRPP. As can be seen, the volume fractions of the rolling texture components sharply decrease with increased TRPP for the asymmetric ratio of 1:2. Nevertheless, they remain higher than the fractions of shear texture components, even at 50% TRPP for the ratio 1:2. Here, among the prominent rolling texture components, the rotated cube component {001}<011> of the α-fiber and the f2 (111)[$\bar{1}\bar{1}$2] component of the γ-fiber emerge as the major texture components. Meanwhile, comparing the shear texture components at 50% TRPP with other lower TRPPs at the same asymmetric ratio, it is clear that their volume fractions increased. It can be also noted in Figure 4a that the volume fractions of the f1 and f2 texture components are no longer equal under asymmetric rolling conditions. Under symmetric rolling conditions, these components are nearly equal. In case of symmetric rolling, most of the rolling texture components show small dependence on the TRPP. Exception is the rotated cube component {001}<011>, which strengthens steadily with the increase in thickness reduction per pass.

#### 3.1.2. High Strain Range (89%)

**Figure 5:**
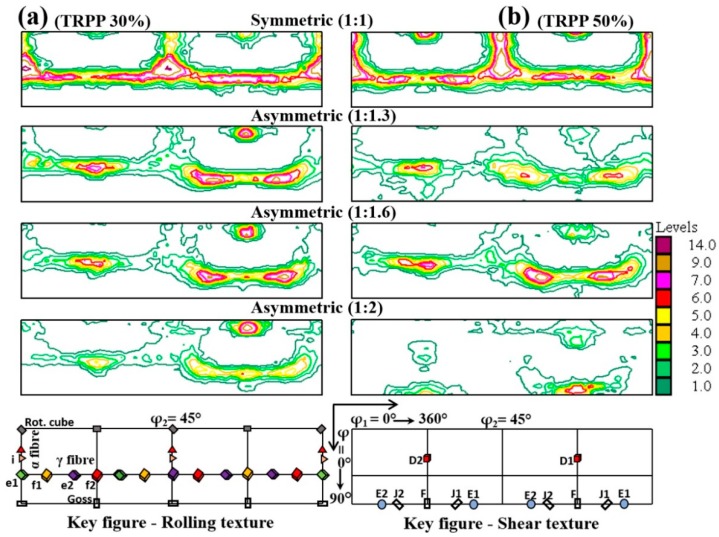
φ_2_ = 45° ODF sections in high strain range (89%) for symmetric and asymmetric rolling with TRPP of (**a**) 30% and (**b**) 50% for varying roll diameter ratios from 1:1 to 1:2.

The φ_2_ = 45° sections of ODFs are plotted in Figure 5 for both TRPP; 30% and 50%, while varying the roll diameter ratio from symmetric (1:1) to extreme asymmetric (1:2) rolling. There are more changes in the ODFs for this large strain case for both symmetric and asymmetric rolling compared to the lower rolling reduction. For symmetric rolling, the α and γ fibers are strong for both 30% and 50% TRPP. With increasing asymmetric rolling, these fibers are much weaker and appear in more and more rotated positions (the rotation of the γ fiber can be best seen in the second half of the ODF; from φ_1_ = 180° to 360°). The rotation is very pronounced for the roll diameters of 1:1.6 and 1:2. The texture is nearly a shear texture for the maximum asymmetry case. 

**Figure 6:**
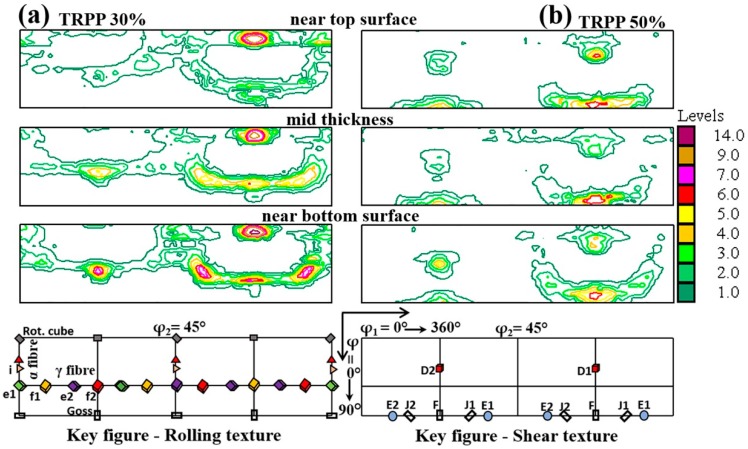
φ_2_ =45° ODF sections at three locations across the thickness of the sheet for high strain (89%) asymmetric rolling (1:2) for TRPP of (**a**) 30% and (**b**) 50%.

It is also important to emphasize that there is a good through-thickness homogeneity of the textures at this strain level for the asymmetric ratio of 1:2 for the 50% TRPP, see Figure 6. For 30% TRPP, the texture is stronger near the bottom surface of the sheet (at the contact with larger roll).

**Figure 7:**
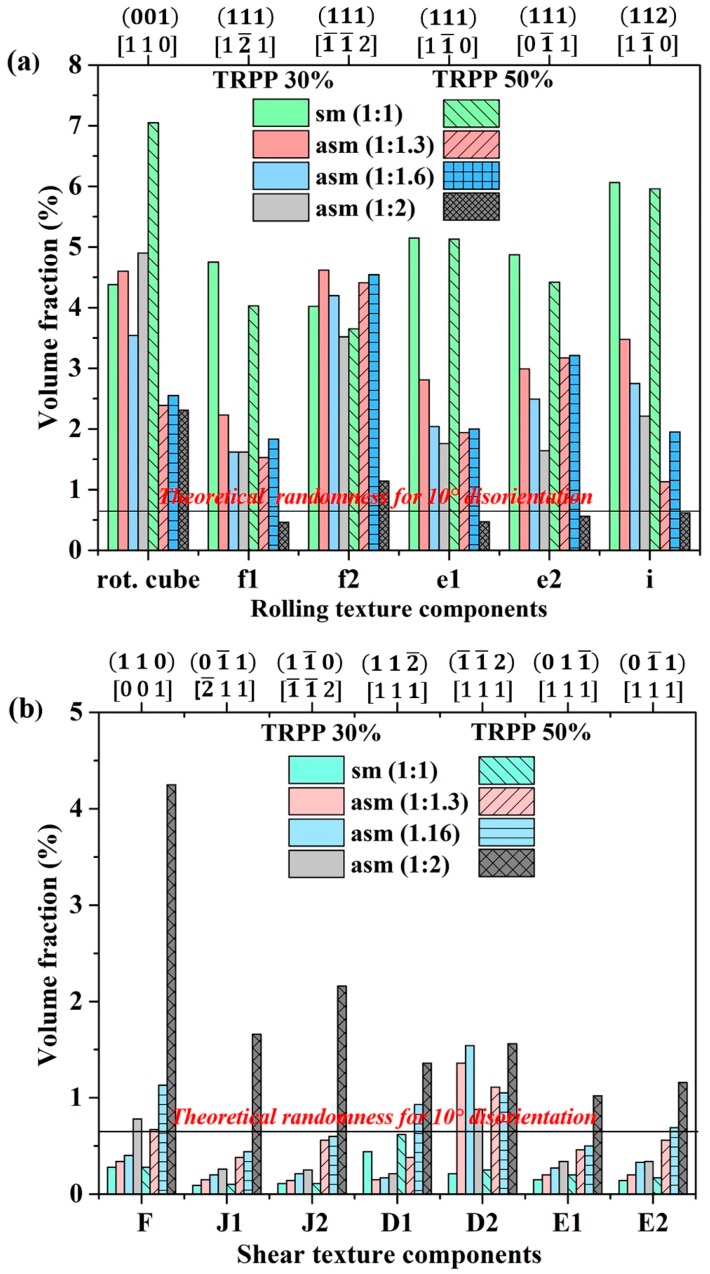
Prominent texture components of (**a**) rolling and (**b**) shear at mid-thickness of the sheet for varying roll diameter ratio from 1:1 to 1:2 at TRPP 30% and 50%.

The computed quantitative volume fraction results presented in Figure 7a,b effectively confirm the general weakening of the rolling texture components with the simultaneous strengthening of the shear texture components for 30% and 50% TRPP. A major rise can be observed for the asymmetry ratio of 1:2, where the volume fractions of the shear texture components jump well above the random intensity level (Figure 7b). 

**Figure 8:**
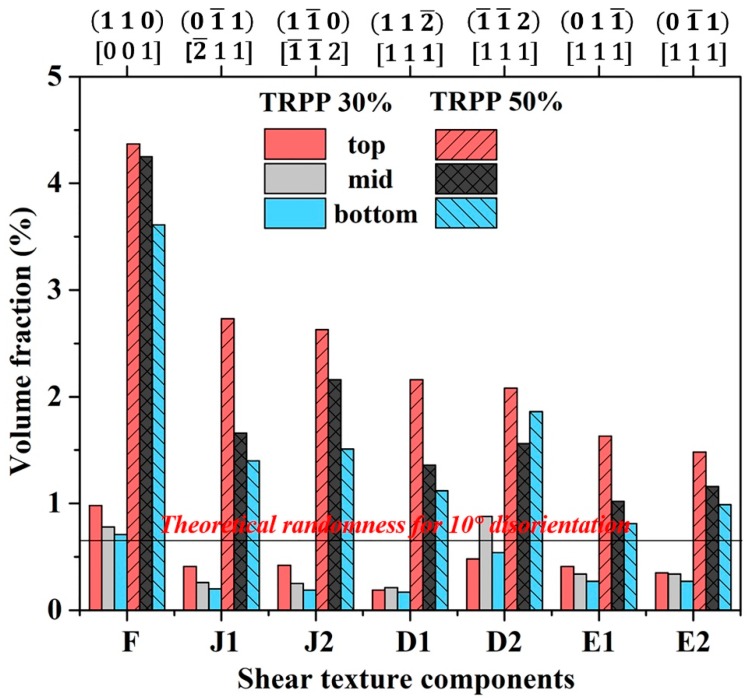
Prominent shear texture components at three locations across the sheet thickness for the asymmetric ratio 1:2 with 30% and 50% TRPP.

Concerning the homogeneity of the shear texture components across the thickness, we can see that their strengths are essentially similar throughout the thickness for the asymmetry ratio 1:2 for both 30% and 50% TRPP (Figure 8). It is also evident from Figure 8 that the strengthening in shear texture components is greater for TRPP of 50% than for 30%.

### 3.2. Microstructure

#### 3.2.1. Medium Strain Range (75%)

**Figure 9:**
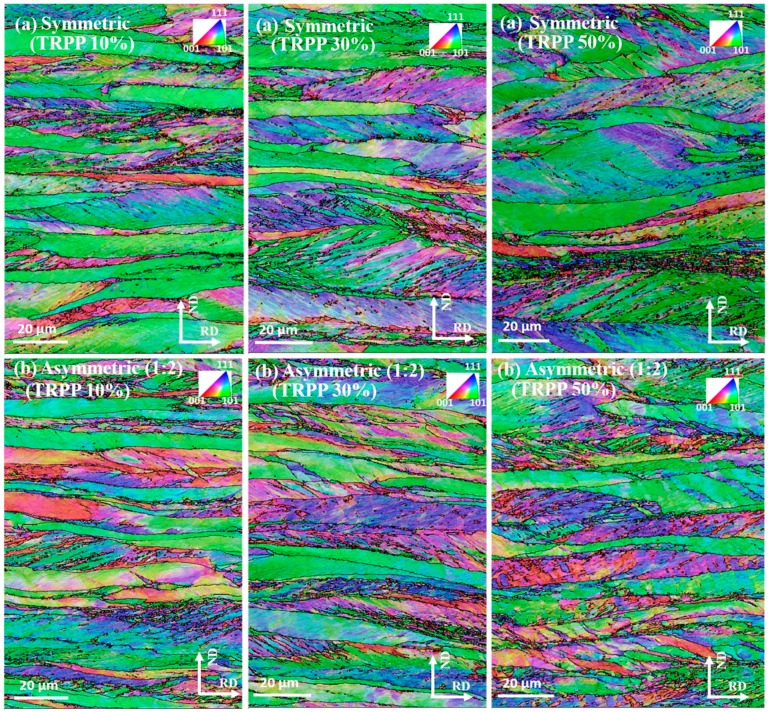
IPF maps (RD axis projection) for medium strain (75%) in (**a**) symmetric and (**b**) asymmetric (1:2) mode with TRPP values of 10, 30, and 50%.

Representative IPF maps of the microstructure taken from the mid-thickness on TD plane can be seen in Figure 9 for both the symmetric and asymmetric (1:2) cases. As can be seen, the grains show their usual tendency of becoming flattened for all TRPP values. The IPF maps at the asymmetry ratio 1:2 show that the fragmentation of the flattened grains increases with increasing TRPP. Additionally, it appears from Figure 9 that the original grain structure is less fragmented in symmetric compared to asymmetric rolling when increasing the TRPP from 10% to 50%.

**Figure 10:**
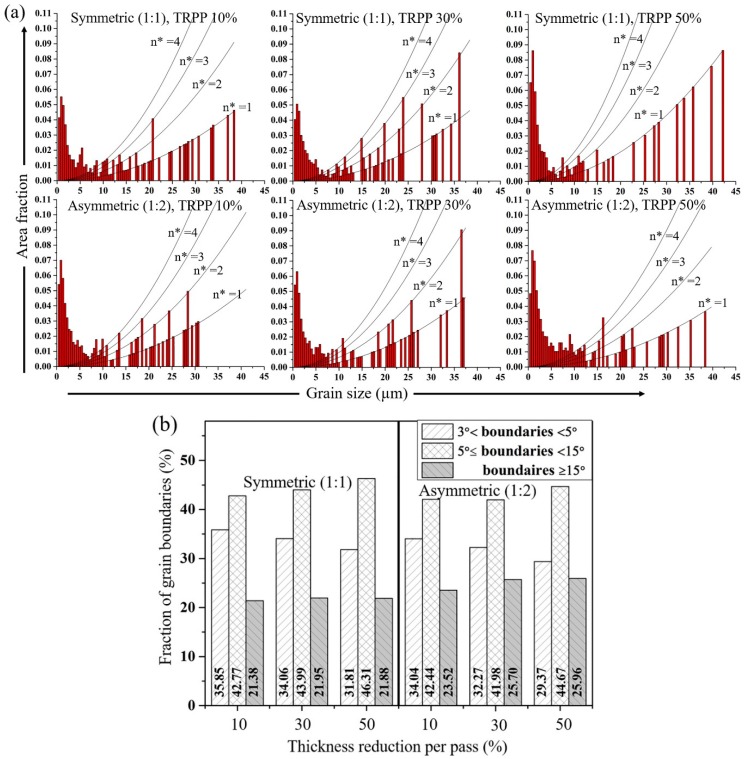
(**a**) Grain size distributions (area fractions) and (**b**) fraction of grain boundaries for symmetric and asymmetric (1:2) rolling in medium strain (75%) for TRPP of 10, 30, and 50%. The added parabolas indicate the number of large grains being in the same bins (n*).

Similar observations can be made in the grain size distributions, see Figure 10a, where the fraction of large grains is greater for the symmetric compared to the asymmetric rolling as TRPP increases to 50%. Clearly, the grain size distributions are bimodal, in all cases. There are many small grains that are below 10 µm size. The large grains size parts of the distribution show parabolic type distributions, which is due to the relatively small numbers of grains found within the selected bin-size of 1 µm [35]. The number of grains corresponding to the different parabolas are indicated in Figure 10a. Another important characteristic of grain fragmentation is the fraction of large angle boundaries. They are illustrated in Figure 10b for three interval values of the misorientations: from 3–5°, 5–15°, and above 15°. As can be seen, the fraction of boundaries having misorientation angles larger than 15° increased more in asymmetric rolling than in the symmetric case, for all TRPP values. 

#### 3.2.2. High Strain Range (89%)

**Figure 11:**
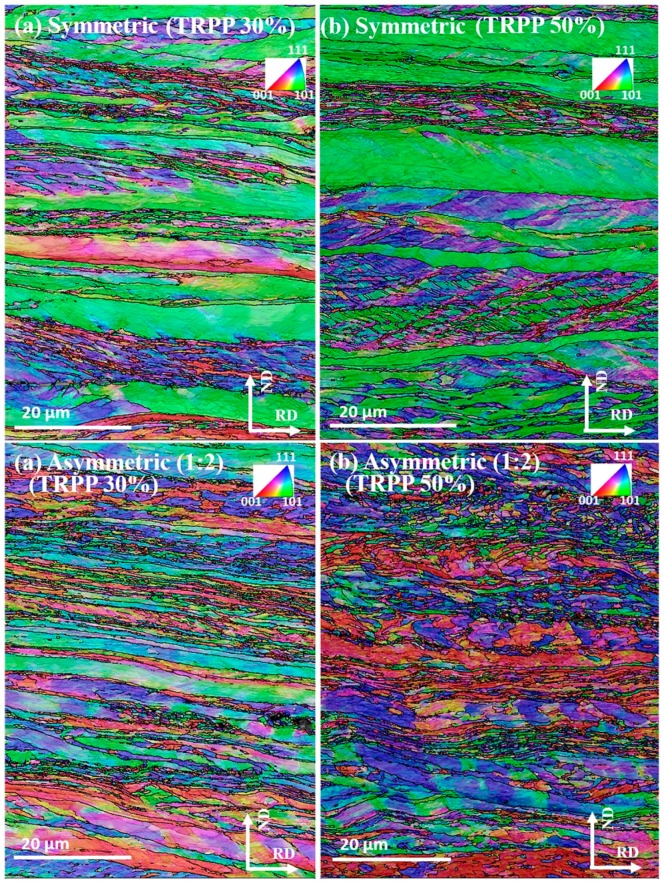
IPF maps (RD axis projection) for high strain (89%) rolled in symmetric and asymmetric (1:2) mode with TRPP of (**a**) 30% and (**b**) 50%.

At 89% thickness reduction for 30% TRPP, it can be seen from Figure 11 via the green colored grains that the mid-thickness microstructure mainly consisted of elongated grains oriented within the α fiber for both symmetric and asymmetric rolling. However, apart from these α grains, high occurrence of blue colored, severely strained areas are evident, where shear bands and deformation bands intersect; these are γ fiber grains for the asymmetry ratio of 1:2. Additionally, it can also be observed at this stage in the IPF maps that the inclination angle of the shear bands to the rolling direction is smaller as compared to the medium strain range for both symmetric and asymmetric rolling. 

A significant change in the rolled microstructure can be observed at the asymmetric ratio of 1:2 for 50% TRPP compared to the symmetric case (Figure 11). Indeed, there is more evidence for grain fragmentation. After 89% reduction in asymmetric rolling, the grains are clearly fragmented for both 30% and 50% TRPPs (Figure 11) but it appears that the orientation gradients are greater in the latter case. For symmetric rolling, the microstructures at 89% reduction changed little even when the TRPP was increased from 30% to 50% (Figure 11), although a higher occurrence of fine grain formation can also be seen in severely deformed regions. This is basically similar to the asymmetric case.

**Figure 12:**
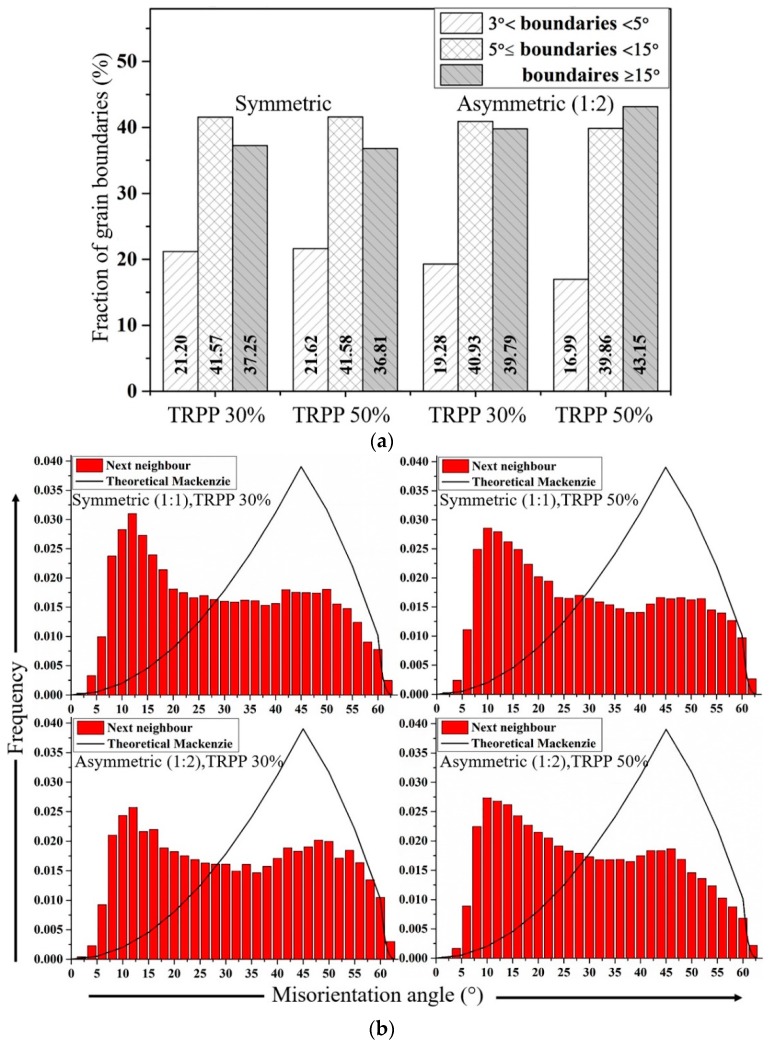

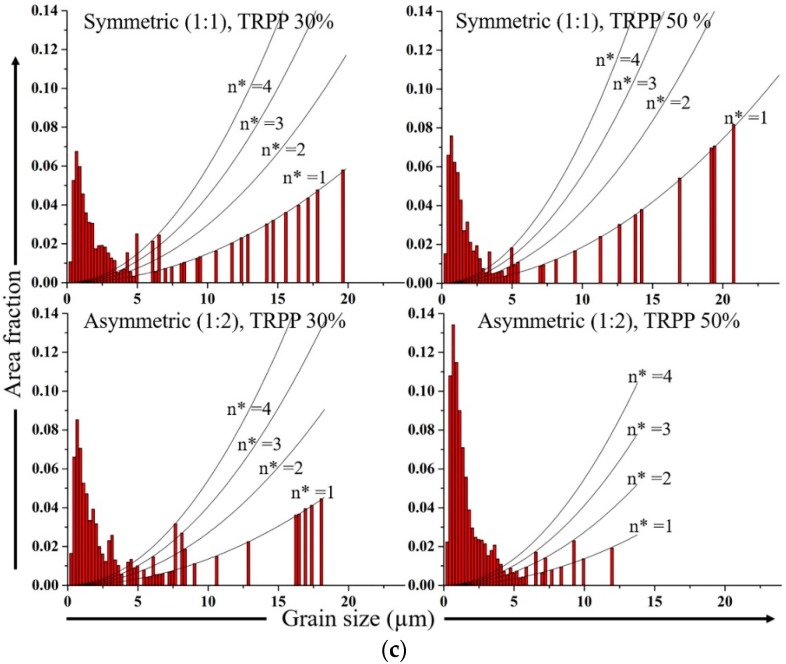
(**a**) Grain boundaries frequencies for symmetric and asymmetric (1:2) rolling for of 30% and 50% in high strain regime (89%); (**b**) next neighbor misorientation distributions of symmetric and asymmetric (1:2) rolling for TRPP of 30% and 50% in the high strain regime (89%); (**c**) grain size distribution (area fraction) for symmetric and asymmetric (1:2) rolling with TRPP of 30% and 50% in high strain regime (89%). The added parabolas indicate the number of large grains being in the same bins (n*).

Figure 12 presents the distributions for grain boundary fractions (Figure 12a), misorientations (Figure 12b) and for normalized average grain sizes (by area fraction method) (Figure 12c). The grain boundary misorientation fraction values in Figure 12a demonstrate that the high angle boundaries in asymmetric rolling are present in significantly higher fraction with respect to the symmetric rolling case. Consequently, the lower misorientations show lower frequency. Interestingly, the next-neighbor grain-to-grain misorientation distributions are very similar, see Figure 12b. This is not in contradiction with the results of Figure 12a because this distribution is not weighed by the grain size. Indeed, there are further differences in the grain size distribution displayed in Figure 12c, where it is clear that the bimodal nature of the distributions is very much reduced for the asymmetric case.

## 4. Discussion

The purpose of the present work is to examine the effects of three parameters—one by one, and also combined—on the texture and microstructure developments during asymmetric rolling of steel. These parameters are: the degree of asymmetry defined by the roll diameter ratios, the thickness reduction per pass (TRPP), and the total rolling reduction (75% and 89%). In the following we discuss the obtained results in terms of the evolution of the crystallographic textures and the characteristics of the microstructures. 

### 4.1. Texture

#### 4.1.1. Relation between Rolling and Shear Textures

First we examine the possible relation between the two kinds of textures that were identified in our experiments: the rolling and shear textures. It will be shown that the shear texture can be derived from the rolling one by a simple rotation. 

Several previous studies show that the main effect on the texture during asymmetric rolling is a general rotation of the rolling texture in the direction of the shear generated by the asymmetry [9,15,16,17,18,19,22]. The velocity gradient of simple shear $\underset{=}{L}$ is composed of the strain rate tensor $\dot{\underset{=}{\varepsilon }}$ and the rigid body spin $\dot{\underset{=}{\beta }}$ according to the decomposition

(1) $$\underset{=}{L}=\left(\begin{array}{ccc} 0 & 0 & \dot{\gamma }\\ 0 & 0 & 0\\ 0 & 0 & 0 \end{array}\right)=\dot{\underset{=}{\varepsilon }}+\dot{\underset{=}{\beta }}=\left(\begin{array}{ccc} 0 & 0 & \dot{\gamma }/2\\ 0 & 0 & 0\\ \dot{\gamma }/2 & 0 & 0 \end{array}\right)+\left(\begin{array}{ccc} 0 & 0 & \dot{\gamma }/2\\ 0 & 0 & 0\\ -\dot{\gamma }/2 & 0 & 0 \end{array}\right)$$

Here the plane of shear is the rolling plane (ND = the plane perpendicular to axis 3), the shear direction is in the rolling direction (RD = axis 1) and $\dot{\gamma }$ is shear strain rate. In the following, we examine only the effect of the rigid body rotation part, which is represented by the second matrix on the right side of Equation (1). This matrix describes a simple rotation around TD (axis 2) with the rotation rate

(2) $$\dot{\omega }=\dot{\gamma }/2$$

This relation implies a simple relation between the shear strain and the rigid body rotation

(3) γ=2ω

**Figure 13:**
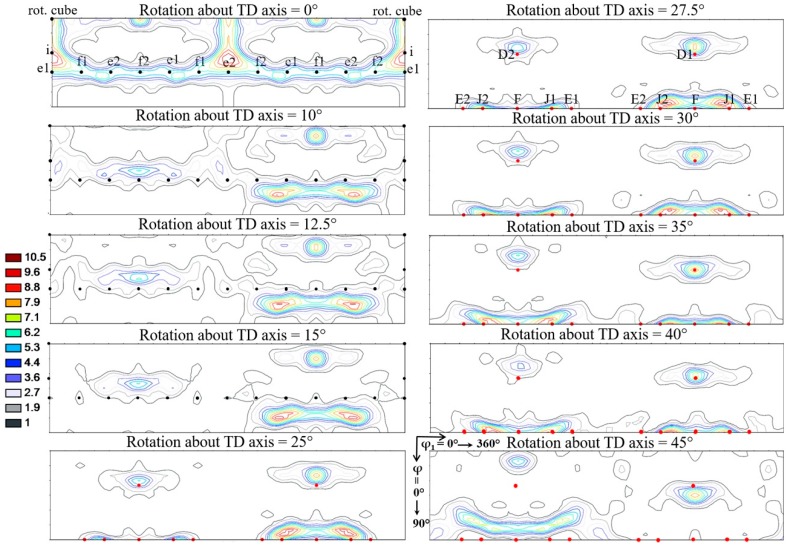
φ_2_ = 45° sections of ODFs to represent the symmetric rolling texture migration towards shear texture just by a simple rotation about the TD axis.

Simple rigid body rotations were applied on an experimental rolling texture with increasing rotation angle around the TD axis, up to ω = 45°. The resulting textures are displayed in the φ_2_ = 45° ODF section in Figure 13 showing the positions of the ideal shear texture components. It can be seen from the figure that an approximate 35° rotation transforms the rolling texture into a shear texture. The corresponding shear strain can be obtained from Equation (3); it is *γ* = 1.22. This value is near to the one proposed from polycrystal texture simulations in [22], which is *γ* = 1.0. Of course, this analysis is only approximate in the sense that the shear component of the imposed strain, with its rigid body rotation, is decoupled from the rolling strain. During a continuous asymmetric rolling process, the two strain components act at the same time with different amounts. Nevertheless, it is very interesting that a rolling texture appears as a shear texture when rotated by about 35°. This angle is the same as the angle at which shear bands appear during rolling [36,37,38]. Therefore, our finding also means that the textures that belong to shear bands appear as perfect rolling textures in the rolling reference system. Thus, shear bands do not make a different texture from rolling, they have no distinct signature on the rolling texture.

We obtained in the preceding paragraph that a rolling texture turns into a shear texture when rotating by 35° around the TD axis. Then the question arises: into which components do the rolling ideal components rotate? We have examined this question using the data in Table 2 and Table 3, and found the following results:

- The f1 and f2 rolling components are rotating exactly into the F component of the shear texture.
- The i rolling component produces the J1 and J2 shear components.
- The rotated cube produces both the D1 and D2 components of shear.
- The e1 and e2 rolling components are not rotating into any ideal shear component.
- The Goss rolling orientation does not need to be rotated to become stable orientation for shear; it is already the same orientation as the F shear component, so it remains stable for any imposed shear deformation after rolling.

Following the above analysis of the texture rotation due to shear, one can attribute a general rotation angle for a given texture made by asymmetric rolling. For this purpose, we choose the deviation angle of the f2 rolling component from its symmetric rolling position. This component is turning progressively into the F shear texture component during asymmetric rolling. The rotation angle needed for complete transformation of the rolling texture into a shear texture is 35°. The texture rotation angles obtained in this way for the different processing conditions are presented in Figure 2b. As can be seen from the rotation values in the sections of the processing space, they increase as a function of all three variables; the total strain, the TRPP, and the roll diameter ratio. The experimentally obtained maximum rotation angle is 32°, which is nearly as high as the theoretical value (35°) obtained above, so the parameter combination pointed to the vertex of the processing cube presented in Figure 1a is the most effective one in terms of transformation of the rolling texture into a shear texture by asymmetric rolling.

#### 4.1.2. Mixed Nature of the Textures Developing during Asymmetric Rolling

One of the main results of our experimental campaign is that the textures obtained by asymmetric rolling show more similarities to the conventional rolling texture if the TRPP is less or equal to 30%, for all diameter ratios (Figure 3a and Figure 6b). It implies that the plane-strain part of the deformation is more effective than the shear part, even for the high strain regime (89%). Nevertheless, the signature of the shear strain is well visible for 30% TRPP when the asymmetry ratio is 1:2.

After increasing the thickness reduction per pass to 50%, even for medium total thickness reductions (75%), the texture for 1:2 ratio shows 15° rotation at mid-thickness of the sheet, meaning that the superimposed shear is effective and competes well with the plane strain part. However, the amount of deformation is not sufficient to significantly strengthen the shear texture. What is happening is that the rolling texture components weaken considerably, but the shear texture does not become strong (Figure 4). An extension of this deformation condition (50% TRPP and asymmetric ratio 1:2) into the high strain regime (89%) not only rotates the texture further towards the ideal shear texture but also strengthens the shear texture components.

The observed higher strengthening in the F (Goss) {110}<001> orientation in high strain regime, as compared to other shear texture components, like J (Brass) {1$\bar{1}$0}<$\bar{1}\bar{1}$2> and D (Copper) {$\bar{1}\bar{1}$2}<111>, is in agreement with previous studies concerning the occurrence of shear textures in shear based processes [39,40,41,42]. The similarity with these reports indicate that the accumulated shear in asymmetric rolling (1:2) with high total thickness reduction (89%) becomes significantly higher than the plane rolling strain. The formation of Goss (F) preferentially along with the copper (D) {$\bar{1}\bar{1}$2}<111> was also observed in strongly sheared regions of Fe3%Si single crystal sheets [40]. These single crystals were initially rotated cube (001)[110] and cube orientations that were hot rolled to 85% thickness reduction by applying higher TRPPs in an unlubricated condition [40]. The mechanism of this behavior was connected to a rotation about the TDǀǀ[110] axis for the rotated cube orientation.

Similar to the observations of single crystal behavior in [40], the rotated cube orientation is important in the present investigation for the development of shear texture components. It can be noticed from symmetric rolling that, with increase in thickness reduction per pass, the rotated cube orientation consistently becomes stronger than the other rolling texture components. Nevertheless, conventionally, under small thickness reduction per pass (below <30%), the i {112}<110> and e {111}<110> components prefer to strengthen more than the rotated cube and f {111}<$1\bar{2}1$> orientations. An increased strengthening in the rotated cube component with increase in thickness reduction per pass can be correlated with two factors. Primarily, its high stability is due to the four-fold symmetry of symmetric rolling and its tendency to have greater orientation spread around the rolling direction than the normal and transverse directions [43]. However, earlier studies of symmetric rolling also reported that under the influence of shear near the surface regions of the sheet, components like the rotated cube and f have strong affinity to form Goss and other shear texture components by virtue of rotation about the TD axis [38,39,40,44,45,46]. Thereby, simultaneous imposition of higher asymmetric ratio along with high thickness reduction per pass forms favorable condition for the rotation of rolling texture and further strengthening in shear texture components with increase in total thickness reduction.

This kind of behavior in terms of texture rotation with 50% TRPP is also in accord with the elastoplastic FEM modelling studies by Kim et al. [15] and with the convergence–divergence map presented by Toth et al. [22] for asymmetric rolling. It was pointed out in these works that only with a high shear coefficient (≥ 1) the shear texture components can be fully developed. Thus, it seems that asymmetric rolling with a roll diameter ratio of 1:2 and 50% thickness reduction per pass possesses a shear coefficient of ≥1.

A comparison of the (1:2) asymmetric rolling texture at high total thickness reduction (89%) with ECAP of IF steel at room temperature shows for ECAP stronger J {110}<112> and D {112}<111> components than the F (Goss) [47,48,49]. ECAP of IF steel was carried at room temperature for both 90° and 120° die angles in route A, which is a monotonic shearing mode with sudden orientation shifts of the crystal orientations between passes (due to the unavoidable re-insertion of the sample into the die). While in lower strain torsion test, the J {110}<112> component appears sooner than the D {$\bar{1}\bar{1}2$}<111> and F {110}<001> orientations [42]. This difference in texture evolution can be related to the imposed deformation geometry of asymmetric rolling where the strain state depends on the respective contributions of plane strain and shear strain, which controls the crystal rotation. In addition, the thickness of the sample does not remain constant throughout the rolling process, while processes such as ECAP and torsion exert a state of simple shear deformation without any change in the shape and size of the material [42,47,48,49]. This also means that more passes can be applied in those processes (ECAP and torsion) which results in stronger shear textures as compared to asymmetric rolling.

### 4.2. Microstructure

It was observed after the total thickness reduction of 75% (medium strain regime) with TRPP up to the 50% that both symmetric and (1:2) asymmetric rolling form elongated and lamellar bands of deformed grains oriented in the rolling direction (Figure 8). However, in the present experiments, the occurrence of orientation inhomogeneity and fragmentation was more pronounced in the asymmetric case when the TRPP was increased to 50%. Increasing the total thickness reduction to the high strain regime (89%) influences not only the asymmetric but also the symmetric rolled microstructures. It is due to the simultaneous activation of both texture and microstructure mechanisms and their respective evolution when deformation increases from medium to high strain regime [50,51]. Thereby, changing the strain regime from medium to high increased the fraction of HAGB from ~22 to ~37% even in the symmetric case with 30% TRPP (Figure 9b and Figure 11a). Although, the fraction of HAGBs achieved in symmetric rolling with further increase in 50% TRPP is still in the same range as observed with 30% TRPP. In fact, it also lies in the same range as reported by Saha et al. for multi-pass rolling of IF steel with small TRPPs [52]. This result indicates that the fraction of HAGBs cannot be increased significantly by increasing TRPP for a given total thickness reduction in symmetric rolling.

In the case of asymmetric rolling (TRPP 30 to 50% and asymmetry ratio 1:2), an increase in deformation and shear bands as a result of fragmentation and inhomogeneity can be anticipated from the imposed deformation condition. There can be two additional effects, which appear in asymmetric rolling compared to the symmetric case. From a geometrical point of view, an increase in macroscopic instabilities, such as macro shear bands, may result from the imposed shear strain as TRPP increases. Whereby, grains can undergo shearing while their flattening/elongation occurs simultaneously. Such external imposed deformation conditions act in addition to the internal constraints of the polycrystalline material which plays an important role in steels and influences the grain orientation stability [53,54]. This effect assists in creating more orientation spread and deviation from the preferred stable end orientations in agreement with the texture results presented in Figure 3 and Figure 6. Thus, it can lead to more orientation splitting and spread in orientations. The other factor could be the strengthening of the shear texture with extension of total thickness reduction from medium to high strain regime. It has been noted that orientations evolving towards their final preferred texture are prone to create deformation induced high angle grain boundaries (HAGBs) [50]. In addition, it is also reported when the process is ideal shear type then the rate of lattice rotation become higher as compared to the symmetric rolling [55]. The consequence of this difference in lattice rotation rate leads to the increased grain refinement and fraction of HAGB in shear assisted processes [55]. The simultaneous occurrence of these two factors along with the microstructural mechanism could be the possible reason for further subdivision of grains and an increased fraction of HAGB in the asymmetric mode, especially for the 50% TRPP compared to symmetric rolling. 

The effect of increased thickness reduction from 30 to 50% in high strain regime is also reflected in the misorientation and grain size distribution plots for both types of rolling (Figure 12b,c). Similar to the studies of Liu et al. [56] and Hughes et al. [50,51], a decrease in the fraction of misorientations between 5° and 10° was observed rather than a further increase, in both types of rolling, irrespective of the applied TRPPs. According to these authors, a decrease in the misorientation distribution between 5° and 10° is due to the dominance of the removal process of lamellar boundaries over their generation. However, when higher misorientation angles (≥15°) were taken into consideration, asymmetric rolling showed an increased fraction as compared to the symmetric cases. Hughes et al. suggested that an increase in misorientation angles (≥15°), especially beyond 30°, may be due to the preferred texture formation in subdivided grains which become more prominent after high total thickness reductions [50,51]. According to Toth et al., however, the development of misorientations between 15° and 30° derives from the internal subdivisions of grains rather than from their original grain boundaries which predominantly lie in the range 30–60° [57]. It is more notable in asymmetric rolling (1:2) than in symmetric rolling due to their higher grain fragmentation.

With increased total thickness reduction, it is also noticed that along with increased fraction of fine grains (≤5 μm), a notable fraction of grains between 5 μm and 22 μm remains after symmetric rolling, for both 30% and 50% TRPPs (Figure 12c). Such a trend reaffirms that increased TRPP does not play a significant role in grain fragmentation during symmetric rolling. Asymmetric rolling, on the contrary, reduces the fraction of large residual grains and increases the fraction of fine grains smaller than 5 μm as TRPP is raised from 30% to 50% (Figure 12c). Such a different behavior in asymmetric rolling also highlights how the thickness reduction per pass (TRPP) is an important parameter for achieving high grain fragmentation at high total thickness reductions.

The above-presented argumentation clearly shows that the addition of shear deformation to the rolling strain by asymmetric rolling is beneficial for the grain fragmentation process. The more the texture approaches a shear texture, better the grain fragmentation is. This represents a significant advantage of the asymmetric rolling over the symmetric one. Figure 1a illustrates that a shear texture is approached the best if all three processing parameters are as high as possible, which is represented by the diagonal line in Figure 1a.

## 5. Conclusions

In the present work, systematic multiple pass rolling has been carried out on extra-low carbon steel in symmetric and asymmetric modes while varying the TRPP between 10–50%. The evolution of the textures and the microstructures were examined at medium (75%) and high (89%) total thickness reductions. The results of our study lead to the following conclusions:

1 The rotation of rolling texture towards the shear texture at the mid-thickness as well as its through-thickness homogeneity are significantly increased by the asymmetry-induced shear strain yielding from the applied TRPP and the final thickness reduction of the sheet with roll diameter ratios ≥1:1.6.
2 For TRPP values below about 30%, the resultant texture of asymmetric rolling shows a greater similarity with the plane strain rolling texture compared to a conventional shear texture but prominent asymmetric trends exist in the preferred texture components especially in the f1 and f2 orientations ({111}<$\bar{1}\bar{1}$2>) of the rolling texture.
3 The texture after asymmetric rolling (1:2) resembles to an ideal shear texture when the deformation is extended to the high strain regime and for high TRPP such as 50%.
4 It has been shown that an approximately 35° rigid body rotation of the rolling texture around the TD axis transforms a typical symmetric rolling texture into a shear texture. Form this, it has been estimated that the shear component generated by the asymmetry conditions has to be at least 1.2 in order to get a shear texture.
5 The shear texture that can be obtained by asymmetric rolling inherits several texture components from the symmetric rolling texture. A list of them is provided in this study.
6 Asymmetric rolling is advantageous for grain refinement and for the creation of deformation induced HAGBs as compared to symmetric rolling under similar conditions. However, the significance of this advantage depends primarily on the final total thickness reduction and is associated with the degree of rotation of the rolling texture.

## Figures and Tables

**Table 1:** Chemical composition of extra low carbon steel in weight percentage (wt %).

C	Mn	Si	Al	Cr	Ni	Cu	Ti
0.0300	0.15	0.006	0.043	0.020	0.010	0.003	0.001

**Table 2:** Ideal deformation rolling texture components of BCC metals and alloys where (hkl)[uvw] represents rolling plane and rolling direction respectively.

Orientation	Miller Indices (hkl)[uvw]	Euler Angles (°) (φ_1,_ φ, φ_2_)
Rotated cube	(001)[110]	0, 0, 45
f1	(111)[$1\bar{2}1$]	30, 54.74, 45
f2	(111)[$\bar{1}\bar{1}2$]	90, 54.74, 45
e1	(111)[$1\bar{1}0$]	0, 54.74, 45
e2	(111)[$0\bar{1}1$]	60, 54.74, 45
i	(112)[$1\bar{1}0$]	0, 35.26, 45

**Table 3:** Ideal deformation shear texture components of BCC metals and alloys expressed in the rolling reference system with hkl ǀǀ ND and uvw ǀǀ RD and their FCC rolling system equivalents in parentheses.

Orientation	Miller Indices (hkl)[uvw]	Euler Angles (°) (φ_1_, φ, φ_2_)
F (Goss)	(110)[001]	(90, 90, 45), (270, 90, 45)
J1 (Brass)	($0\bar{1}1$)[$\bar{2}11$]	(125, 90, 45), (305, 90, 45)
J2 (Brass)	($1\bar{1}0$)[$\bar{1}\bar{1}2$]	(55, 90, 45), (235, 90, 45)
D1 (Copper)	($11\bar{2}$)[111]	270, 35, 45
D2 (Copper)	($\bar{1}\bar{1}2$)[111]	90, 35, 45
E1	($01\bar{1}$)[111]	(145, 90, 45), (325, 90, 45)
E2	($0\bar{1}1$)[111]	(35, 90, 45), (215, 90, 45)
